# Unaltered Brain GABA Concentrations and Resting fMRI Activity in Functional Dyspepsia With and Without Comorbid Depression

**DOI:** 10.3389/fpsyt.2020.549749

**Published:** 2020-09-11

**Authors:** Arthur D. P. Mak, Yuen Man Ho, Owen N. W. Leung, Idy Wing Yi Chou, Rashid Lui, Sunny Wong, David K. W. Yeung, Winnie C. W. Chu, Richard Edden, Sandra Chan, Linda Lam, Justin Wu

**Affiliations:** ^1^ Department of Psychiatry, Faculty of Medicine, The Chinese University of Hong Kong, Hong Kong, Hong Kong; ^2^ Institute of Digestive Disease, Department of Medicine and Therapeutics, Faculty of Medicine, The Chinese University of Hong Kong, Hong Kong, Hong Kong; ^3^ Department of Imaging and Interventional Radiology, Faculty of Medicine, The Chinese University of Hong Kong, Hong Kong, Hong Kong; ^4^ Russell H. Morgan Department of Radiology and Radiological Science, The Johns Hopkins University School of Medicine, Baltimore, MD, United States; ^5^ F.M. Kirby Research Center for Functional Brain Imaging, Kennedy Krieger Institute, Baltimore, MD, United States

**Keywords:** major depressive disorder, functional dyspepsia, GABA, magnetic resonance spectroscopy, resting fMRI

## Abstract

**Background:**

GABA-deficit characterizes depression (MDD), which is highly comorbid with Functional Dyspepsia (FD). We examined brain GABA concentrations and resting activities in post-prandial distress subtype FD (FD-PDS) patients with and without MDD.

**Methods:**

24 female age/education-matched FD-PDS with comorbid MDD (FD-PDS-MDD), non-depressed FD-PDS, and healthy controls each were compared on GABA concentrations, resting fMRI (fALFF) in bilateral pregenual anterior cingulate (pgACC), left dorsolateral prefrontal cortex (DLPFC), insula, and somatosensory cortex (SSC).

**Results:**

FD-PDS-MDD patients had mild though elevated depressive symptoms. FD-PDS patients had generally mild dyspeptic symptoms. No significant between-group differences in GABA or fALFF were found. No significant correlations were found between GABA and depressive/dyspeptic symptoms after Bonferroni correction. In patients, GABA correlated positively with left insula fALFF (r = 0.38, Bonferroni-corrected p = .03).

**Conclusion:**

We did not find altered GABA concentrations or brain resting activity in FD-PDS or its MDD comorbidity. The neurochemical link between MDD and FD remains elusive.

## Introduction

Functional dyspepsia (FD) is a common and chronic functional gastrointestinal disorder characterized by symptoms attributed to gastroduodenal origin in the absence of any organic, systemic, or metabolic disease likely to explain the symptoms (1).

Substantial evidence exists for gut-brain directed etiological mechanisms in FD, such as infection, inflammatory mechanisms, and altered gut microbiota. On the other hand, associations of FD with personality traits, serotonin polymorphism and experience of childhood adversity provide some evidence for the brain-gut basis to FD (2). In particular, the significantly increased risk of comorbid Major Depressive Disorder (MDD) in community FD samples (3–5), bidirectional chronological relationship in the respective onsets of FD and MDD in community subjects (6), and response of non-depressed FD subjects to serotonin-modulating antidepressant drugs (7, 8) suggested substantial pathophysiological overlap between FD and MDD. In addition, both MDD and FD have been associated with trait characteristics of neuroticism (9, 10) and alexithymia. Alexithymia has even been shown to contribute to both somatization, depressive and anxiety symptoms, as well as suicide risk (11, 12). MDD has also been associated with trait-alterations in sensory processing patterns unique to the individual (13) that is akin to the experience-based alterations in salience computation of visceral signals in functional gastrointestinal disorders (14, 15). Examining neural mechanisms underlying the rich linkages in the FD-MDD comorbidity should help understand the brain-gut mechanisms that determine symptomatology and treatment response.

Visceral, cognitive and emotional processing occurs in cortical glutamatergic networks, including somatosensory cortex (SSC) [Sensorimotor Network (SMN) (15)] that relays visceral sensory input to the Salience Network (SN-insula) which integrates affective and interoceptive information (16) and send control signals to the (i) the Default Mode Network [DMN: midline structures including perigenual anterior cingulate cortex (pgACC)] (16) and (ii) engages the Executive Network [EN: Dorsal-lateral prefrontal cortex (DLPFC)]. These glutamatergic networks are modulated by inhibitory GABA-ergic interneurons (16), in turn innervated by serotonergic neurons at pgACC, VMPFC, and raphe nuclei (17). Dysfunction in these affective networks may lead to impaired gating of somatosensory signals (2, 16), contributing to somatosensory amplification (11, 14) in FD and MDD (18).

In MDD, inhibition of GABA-ergic neurons by dysfunctional serotonergic neurons at the pgACC may reduce GABA (19, 20), in turn disinhibiting glutamatergic neurons and resulting in DMN hyperactivity (21–23). This altered pgACC GABA/glutamate balance may underlie increased self-focus in MDD and has been suggested to be the neurochemical substrate of antidepressant action (24). MDD is also associated with resting-state hypoactivity in the EN (DLPFC) (22, 25) while in the insula, depressive affect is associated with reduced GABA concentration and insula hypoactivity in processing self-related information (26).

In FD-PDS, the predominant FD subtype where psychiatric comorbidity is the commonest, resting-state positron-emission tomography (PET) (27) and functional MRI (28) showed increased SN, DMN, thalamus, and precuneus resting activity, as well as lowered SMN and SN activation threshold to gastric distension and failure of pain-related pgACC activation (29). These correlated positively with dyspeptic (27), anxiety, and depressive symptoms (29), suggesting abnormal mood-related processing of somatic signals in the SMN, SN, and DMN (29–31). In our earlier magnetic resonance spectroscopy (MRS) study, we found significant increase in SSC glutamate levels in FD that correlated with anxiety and dyspeptic symptom severity but were independent of depressive severity (32). The short-TE PRESS sequence used for resolving glutamate peaks from glutamine (TR/TE = 3,000/24ms) did not yield accurate measurements of GABA. In fact, there have been no studies on GABA transmission in FD. As such, it is unknown whether FD-PDS would be similar to MDD in GABA-deficit-driven SN and DMN abnormal activities.

In the present investigation, we used MEGAPRESS (33) to examine GABA concentrations in major nodes of the SN, SMN, DMN, and EN in matched FD-PDS patient groups differentiated by the presence of MDD, to clarify the pathophysiological abnormalities peculiar to FD and depressive comorbidity. To reduce demand on sample size and owing to evidence showing gender effects in neural activities in the brain (34), as well as the female predominance in MDD and FD (32), we decided to perform the study on female subjects only. We hypothesized that altered GABA concentrations in representative regions in the DMN, SN, EN, and SMN would be found amid subjects with FD-PDS with and without comorbid MDD as compared to healthy controls, with those with comorbid MDD showing a greater deficit than their counterparts.

## Materials and Methods

### Participants and Recruitment

Twenty-six FD-PDS with comorbid MDD (FD-PDS-MDD) and 28 FD-PDS without comorbid MDD were consecutively recruited at a specialist gastrointestinal clinic. Inclusion criteria were as follows: (i) currently satisfying ROME III criteria for FD-PDS as assessed by a gastroenterologist (1), (ii) normal oesophago-gastroduodenoscopy results within 2 years from recruitment, (iii) for FD-PDS-MDD group only, comorbid Major Depressive Disorder (MDD) as meeting DSM-IV-TR criteria for MDD, and (iv) right-handed. Exclusion criteria included having other functional gastrointestinal disorders (irritable bowel syndrome or history of symptoms of acid regurgitation, heartburn or those with abdominal pain as the predominant symptom), consuming drugs that may affect gastrointestinal motility (neuroleptic drugs, non-steroidal anti-inflammatory drugs, aspirin and steroids) within 2 weeks prior to scan, chronic or severe medical conditions, intellectual disability, organic brain syndromes, having other mental disorders including anxiety disorders, psychosis, bipolar disorder, substance abuse, or dependence and presence of any metallic devices or materials in the body that are contraindicated for magnetic resonance imaging.

Thirty age-sex-education matched healthy controls (HC) were recruited *via* hospital posters and online advertisements, after a baseline assessment screening against the same exclusion criteria described above, along with any personal history of gastrointestinal complaints and personal or family history of any mental disorders.

Ten subjects with excessively noisy MRS spectra were excluded from analyses, resulting in a final sample consisting of 24 subjects per arm.

All eligible participants provided valid written informed consent. All experimental protocols were approved by the New Territories East Cluster-Chinese University of Hong Kong Clinical Research Ethics Committee and all procedures were performed in accordance with the approved guidelines and regulations.

### Sociodemographic and Symptomatic Assessments

Socio-demographic data including age, marital status, employment status, monthly family income, and educational background were obtained using a questionnaire. Diagnoses of MDD and other mental disorders stated within the exclusion criteria were obtained by trained clinician interviewers using the Chinese-Bilingual version of the Structured Clinical Interview for DSM IV-TR Mental Disorders-I (35). Current dyspeptic symptoms and other gastrointestinal disorder symptoms were enquired with a structured Rome III symptom assessment questionnaire. Dyspeptic symptom severity was measured with the dyspepsia items from FGI-Checklist, a validated 20-item structured questionnaire assessing common upper and lower gastrointestinal symptoms (36), each self-rated on a scale of 0 to 3. A global dyspeptic symptom severity score was generated by averaging the scores for the items included. Current depressive and anxiety symptoms were evaluated with the commonly-used interviewer-administered scales of MADRS (37) and HAM-A (38), respectively. MADRS consists of 10 items rated on a 0 to 6 continuum that evaluate core symptoms of depression, with a total score of 7–19 denoting mild depression, 20–34 for moderate depression, and >34 for severe depression. HAM-A consists of 14 items on psychic and somatic aspects of anxiety, each rated from 0 (not present) to 4 (severe), with a total score of <17 denoting mild severity, 18–24 for mild to moderate, and 25–30 for moderate to severe. Interoceptive awareness was measured using the Body Perception Questionnaire, a 122-item self-report instrument consisting of subscales on body awareness, stress response, autonomic nervous system reactivity, stress style, and health history, respectively (39). General severity of somatic symptoms was measured using a Chinese version of the 15-item Patient Health Questionnaire (PHQ-15) which has been validated in the local community, showing satisfactory reliability and validity (40). General health-related quality of life was measured by a validated Chinese version of the Short-form-36 health survey (SF-36) (41). Current medication history was enquired and checked with hospital records. The Three-subtest Short Form of the Wechsler Adult Intelligence Scale-III (42) was conducted on all participants at baseline to control for confounding effects of intelligence on scan results.

### Imaging

All imaging protocols were performed using a 3 Tesla MRI scanner (Achieva TX series, Philips Healthcare, Best, Netherlands).

#### Anatomical MRI

High resolution structural images were acquired using an eight-channel receive-only head coil using a sagittal 3D T1-weighted sequence (TR/TE: 7.4/3.4 ms; field of view: 250,250 mm, 285 contiguous slices, 0.6-mm (RL) thickness, reconstruction matrix: 240,240, flip angle 8°). T1-weighted images were segmented into gray matter (GM), white matter and cerebrospinal fluid (CSF) maps using SPM12 (43). GM volume of each MRS volume-of-interest (see below) was extracted using FSL (44).

#### Magnetic Resonance Spectroscopy

**Figure 1:**
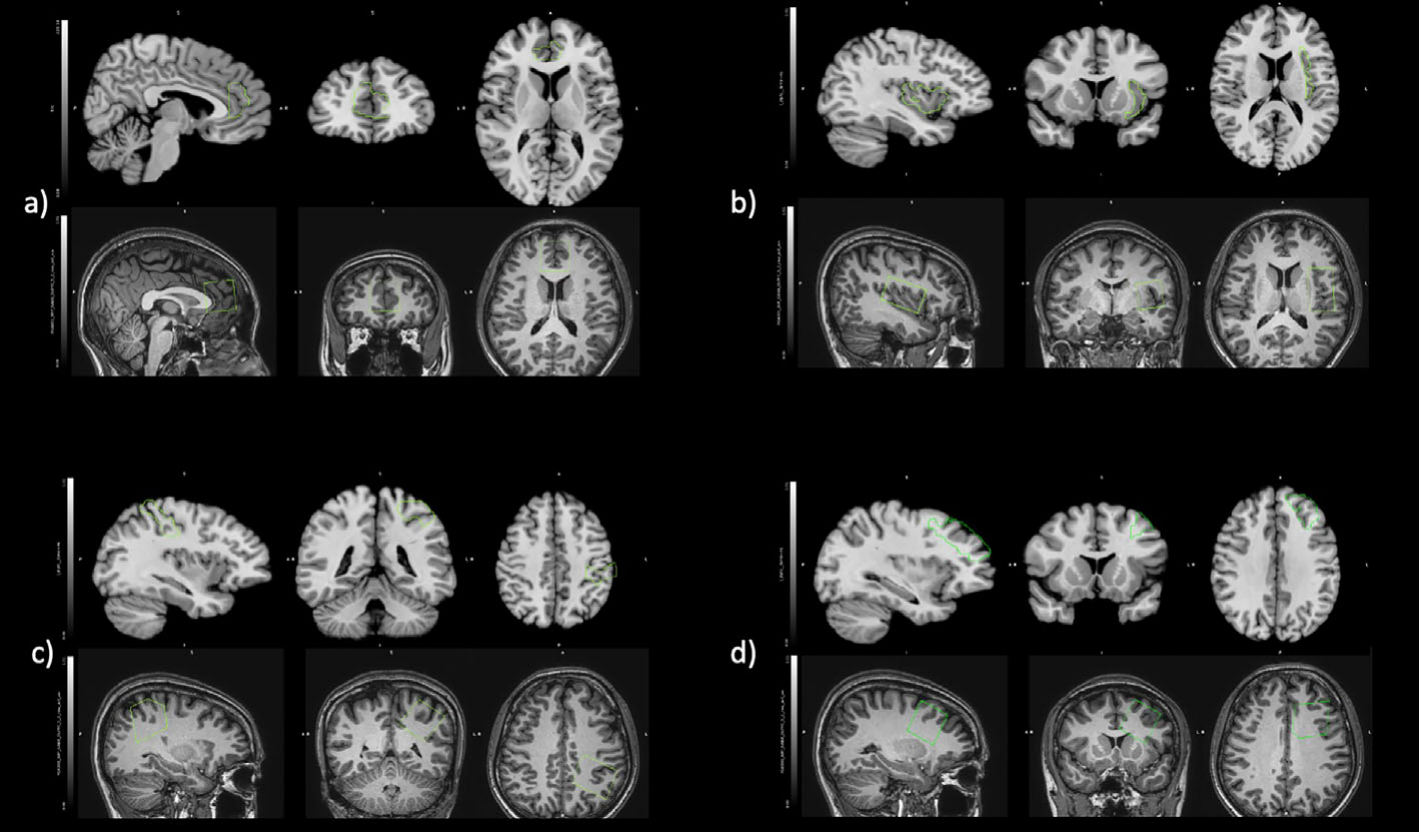
Positioning of regions of interest in fALFF (Top) and MRS (Bottom) for **(A)** bilateral pregenual anterior cingulate cortex, **(B)** left insula, **(C)** left somatosensory cortex, and **(D)** left dorsolateral prefrontal cortex.

Anatomical MRI images were used to guide positioning of MRS volumes-of-interest in bilateral pregenual anterior cingulate cortex (pgACC), left insula, left SSC, and left dorsolateral prefrontal cortex (DLPFC). The bilateral pgACC voxels (30 mm × 30 mm × 30 mm) were positioned bordering the lower edge of the genu of the corpus callosum and with its posterior limits just touching the anterior border of the genu of the corpus callosum (**Figure 1A**) (45). The left insula voxels (25 mm × 40 mm × 25 mm) were aligned along the edge of the insula cortex in an anterior-posterior direction with the anterior edge of the volume-of-interest aligned to the anterior limit of the insula (**Figure 1B**) (26). The left SSC (30 mm × 30 mm × 30 mm) was placed on the post-central gyrus (Brodmann areas 1, 2, and 3) (**Figure 1C**) (46). The left DLPFC (30 mm × 30 mm × 30 mm) was placed in the left inferior frontal gyrus between the inferior frontal sulcus and horizontal ramus (**Figure 1D**) (47, 48).

Signal reception for H-MRS were achieved using the same eight-channel received-only head coil under the following parameters: TE, 68 ms; TR, 2,000 ms; 400 transients of 4,096 data points acquired in 12 min; 14-ms Gaussian editing pulses to be applied either to the GABA spins (at 1.9 ppm) or symmetrically about the water peak (7.5 ppm) in an interleaved manner. A further eight transients were acquired, without water suppression, as an internal concentration reference.

GABA concentrations in the 4 volumes-of-interest were gauged as GABA signal intensities relative to water, as derived from GABA-edited MEGA-PRESS MRS spectra using Gannet v3.0 (33). GABA signal was scaled to account for the fraction of cerebrospinal fluid (CSF) within the voxel, and the water signal was scaled to account for the different water content in CSF, gray, and white matter. A concentration measurement in institutional units is derived from the ratio of the GABA and water signals by further adjusting for the editing efficiency and the T1 and T2 relaxation times of water and GABA. The overall fit errors, represented by the standard deviation of the fitting residuals relative to the GABA and water fitting peaks, converged to mean values of 6.5%, 5.1%, 5.3%, and 4.1% for left DLPFC, left insula, pgACC, and left SSC, respectively.

#### Functional MRI - fALFF

Subjects were instructed to have their eyes closed for 6 min and hold still without falling asleep. Resting fMRI data was collected with a single-shot echo-planar sequence sensitive to BOLD contrast (TR, 2,050 ms; TE, 25 ms; flip angle, 90°; slab thickness, 150 mm; field of view, 205’ 205 mm; image matrix, 6,464; parallel imaging acceleration factor of 2) to acquire 150 dynamic scans under each condition. BOLD images acquired had a nominal in-plane resolution of 3.23.2 mm and a temporal resolution of 2 s per dynamic scan.

The first three volumes were removed to eliminate the effects of start-up transients. Resting-state imaging data were preprocessed using AFNI (Analysis of Functional Neuro-Images) (49). Preprocessing steps included slice-timing correction, alignment between the structural image and the third functional volume, estimation of motion correction parameters (three translational and three rotational components) that co-register each functional volume to a reference volume with the lowest motion, and warping structural image to the Talaraich space. Fractional amplitude of low frequency fluctuations (fALFF) were derived from the rsfMRI data using AFNI’s 3dRSFC program (50, 51). First, each voxel time series was converted into the frequency domain (0–0.25 Hz) using a fast Fourier transform without band-pass filtering and the square root of each frequency of the power spectrum was calculated. Second, each voxel time-series was band-pass filtered (0.01–0.10 Hz) before converting to the power spectrum and the sum amplitude of this low frequency domain was calculated. Finally, the ratio between the sum amplitude of the low frequency domain and the full frequency domain was extracted for each voxel. Also, mean fALFF was calculated for each volume-of-interest.

### Statistical Analysis

Baseline demographic characteristics were compared between the three arms using chi-square and one-way ANOVAs as appropriate.

Mean GABA concentrations were compared between the three groups using generalized estimating equations, ANCOVA, and a t-test where healthy controls were compared against a combined patient group (FD with comorbid MDD + pure FD). Generalized estimating equation analyses were all adjusted for age, IQ, and same-region GM volume, whereas ANCOVA and t-test included any of these covariates only when correlation with GABA concentration was presented.

Pearson correlation was used to identify relationships between GABA concentrations of each region with dyspeptic severity (FGISQ), introspective awareness (BDQ), depressive severity (MADRS), anxiety severity (HAMA), somatization (PHQ15), health-related quality of life (SF36), and same region fALFF. For each VOI, the mean fALFF was extracted and its correlation with same-region GABA concentrations was examined by Pearson correlation, corrected for age, IQ and same-region GM volume, or any combination therefor, whichever correlated with GABA concentration. All correlation analyses were performed across the whole sample, for each patient group individually, and for a combined patient group (FD-PDS and FD-PDS-MDD).

Between group mean difference of fALFF was assessed using voxel-wise permutation analysis, corrected for the effect of age, IQ and same-region GM volume.

IBM SPSS Statistics v23 for Windows was used for all statistical analyses. Results of p < 0.05 were considered significant. All tests were two-tailed. To account for multiple comparisons in four ROIs, Bonferroni correction was applied to all statistical analysis involving GABA and fALFF.

## Results

### Demographics

Twenty-four female subjects were recruited in each group: 1. FD-PDS patients with comorbid MDD (FD-PDS-MDD), 2. FD-PDS patients without MDD (FD-PDS), and 3. healthy controls (HC). 10 subjects with noisy MRS spectra were excluded (see *Materials and Methods*). All subjects were female and had a mean age of 42.44 and on average 11.86 years of education. Over half of the sample were married and had full-time employment. No significant differences were found in age, education, marital status, and employment status between the 3 groups (**Table 1**).

**Table 1:** Demographics.

	FD-PDS-MDD	FD-PDS	HC	Chi-square	F/W	p	Cohen’s d			Hedges’ g
	(n = 24)	(n = 24)	(n = 24)				FD-PDS-MDD	FD-PDS-MDD	FD-PDS vs. HC	All patients vs. HC
							vs. FD-PDS	vs. HC		
**Sociodemographic**										
Age, years (SD)	41.92 (10.88)	42.75 (11.96)	42.67 (12.13)	–	0.04	0.96	–	–	–	–
Education, years (SD)	11.78 (2.63)	11.75 (2.25)	12.04 (2.33)	–	0.11	0.9	–	–	–	–
Marital status, n (%)				9.46	–	0.49	–	–	–	–
** **Single	7 (30.4)	8 (33.3)	10 (41.7)	–	–	–	–	–	–	–
** **Married	11 (47.8)	14 (58.3)	13 (54.2)	–	–	–	–	–	–	–
** **Separated/widowed	4 (17.3)	2 (8.3)	1 (4.2)	–	–	–	–	–	–	–
** **Others	2 (8.3)	0 (0.0)	0 (0.0)	–	–	–	–	–	–	–
Occupation status, n (%)^a^				3.04	–	0.8	–	–	–	–
** **Employed	17 (70.8)	16 (66.7)	20 (83.3)	–	–	–	–	–	–	–
** **Student	3 (12.5)	3 (12.5)	2 (8.3)	–	–	–	–	–	–	–
** **Homemaker	3 (12.5)	5 (20.8)	2 (8.3)	–	–	–	–	–	–	–
** **Missing data	1 (4.2)	0 (0.0)	0 (0.0)	–	–	–	–	–	–	–
**Affective Symptoms** ^a^										
Depression—MADRS	18.08 (6.08)	1.71 (2.14)	0.67 (1.24)	–	93.21	<.001^***^	3.59^***^	3.97^***^	0.60^*^	1.19^***^
Anxiety—HAMA	15.38 (7.10)	5.29 (4.79)	2.17 (2.30)	–	38.44	<.001^***^	1.67^***^	2.50^***^	0.83^**^	1.24^***^
Somatization—PHQ-15	12.52 (3.96)	7.54 (2.64)	2.96 (2.81)	–	46.67	<.001^***^	1.48^***^	2.78^***^	1.68^***^	1.86^***^
**Dyspeptic Symptoms – FGI-Checklist** ^a^										
Symptom severity^b^	4.48 (2.13)	3.42 (1.44)	0.50 (.88)	–	57.62	<.001^***^	0.58	2.44^***^	2.44^***^	2.13^***^

a Equal variances are not assumed.

b Composite score from summing self-rated severity of four symptoms – egipastric pain, egipastric burning, postprandial fullness, early satiation, each rated on a four-point scale of 0 to 3, i.e., 0 (none), 1 (mild), 2 (moderate), and 3 (severe).

*p < 0.05, **p < 0.01, ***p < 0.001.

### Clinical

FD-PDS-MDD patients had a mean Montgomery–Åsberg Depression Rating Scale (MADRS) and Hamilton Anxiety Rating Scale (HAMA) scores of 18.01 (SD 6.08) and 15.38 (SD 7.1), which were within the mild range (37, 38). These were significantly greater than the FD-PDS group (ps <.001) and HC (ps < .001) (**Table 1**).

Patients had generally mild dyspeptic symptoms. On a composite score of current dyspeptic symptom derived by summing self-rated scores on four dyspeptic symptoms (36) (epigastric pain, epigastric burning, postprandial fullness, early satiety), FD-PDS patients with and without MDD comorbid showed significantly elevated dyspeptic symptoms compared to HC (p < 0.001), while no significant differences were found between FD-PDS and comorbid FD-PDS-MDD patients (**Table 1**).

### Group Difference in GABA Concentration and fALFF

**Figure 2:**
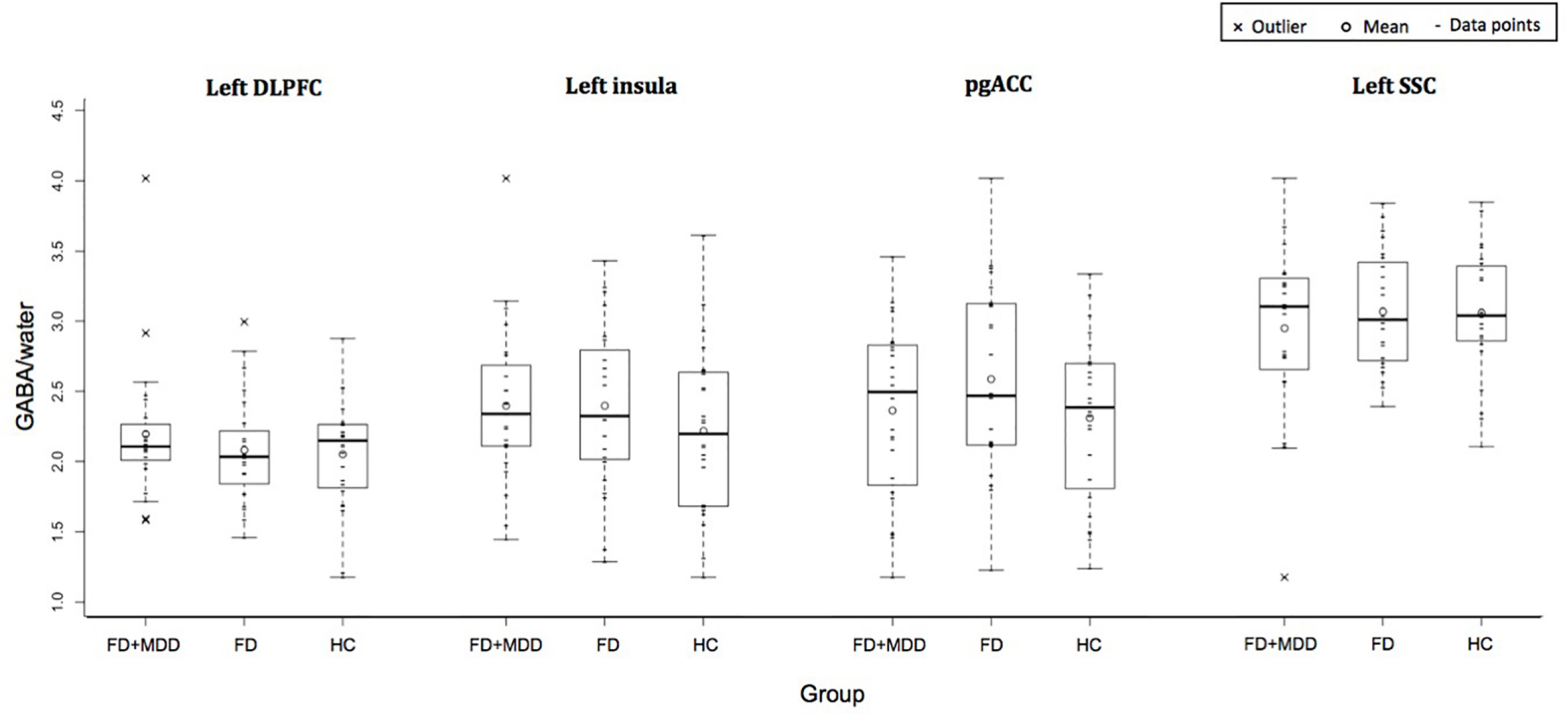
Group difference in GABA/water in four regions of interest: left dorsolateral prefrontal cortex, left insula, bilateral pregenual anterior cingulate cortex, and left somatosensory cortex.

**Figure 2** shows the GABA concentration in different VOIs in the three groups. No significant differences were found between the three groups in GABA concentrations in any of the voxels of interest, using AN(C)OVA (**Table 2**), generalized estimating equation (**Table 2**), or in the T-test comparing HC against a combined patient group (**Table 3**).

**Table 2:** Group difference of GABA/water.

AN(C)OVA																			
			FD-PDS-MDD				FD-PDS			HC			AN(C)OVA^a^						
			(*n* = 24)				(*n* = 24)			(*n* = 24)							
ROI			mean		SD	mean			SD	Mean		SD	F	df	p	Bonferroni-corrected p			
Left Dorsolateral Prefrontal Cortex^b^			2.2		0.49	2.08			0.38	2.05		0.39	1.7	2, 1, 68	0.19	0.76			
Left Insula			2.53		0.22	2.53			0.23	2.46		0.24	0.8	2, 69	0.46	>0.99			
Bilateral Pregenual Anterior Cingulate			2.3		0.23	2.38			0.25	2.28		0.22	1.3	2, 69	0.28	>0.99			
Left Somatosensory Cortex			2.28		0.33	2.34			0.22	2.33		0.23	0.4	2, 69	0.67	>0.99			
**Generalized Estimating Equation^c^**																			
**ROI**	**Group**	**β**		**Z**			**p**	**Bonferroni-corrected p**			**95% CI**								
Left DLPFC	FD-PDS-MDD																	
	FD-PDS	-0.16		-1.26			0.21	0.84			0.85 (0.67-1.09)								
	HC	-0.21		-1.63			0.1	0.4			0.81 (0.63-1.04)								
Left Insula	FD-PDS-MDD																		
	FD-PDS	0.002		0.03			0.98	>0.99			1 (0.89-1.13)								
	HC	-0.07		-1.02			0.31	>0.99			0.94 (0.82-1.06)								
pgACC	FD-PDS-MDD																		
	FD-PDS	0.1		1.41			0.16	0.64			1.1 (0.96-1.27)								
	HC	0.003		0.04			0.97	>0.99			1 (0.88-1.14)								
Left SSC	FD-PDS-MDD																		
	FD-PDS	0.08		1.03			0.3	>0.99			1.09 (0.93-1.28)								
	HC	0.1		1.1			0.27	>0.99			1.1 (0.93-1.31)								

^a^Levene's test confirmed equal variances across groups.

^b^Covariate: IQ.

^c^Adjusted for age, gray matter volume in same-region MRS ROI and IQ.

**Table 3:** Group difference of GABA/water between healthy controls and combined patient group tested with t-test (n = 72).

ROI	All patients (n = 48)		HC (n = 24)		t-test^a^			
	mean	SD	mean	SD	t	df	p	Bonferroni-corrected p
Left Dorsolateral Prefrontal Cortex	2.14	0.44	2.05	0.39	-0.8	70	0.2	0.8
Left Insula	2.53	0.22	2.46	0.24	-1.3	70	0.11	0.44
Bilateral Pregenual Anterior Cingulate	2.34	0.24	2.28	0.22	-1	70	0.15	0.6
Left Somatosensory Cortex	2.31	0.28	2.33	0.23	0.4	70	0.35	>0.99

^a^No significant correlation present in GABA/water of individual ROI against age, same-region gray matter volume and IQ.

No significant difference was found in fALFF among the three groups in any of the four regions examined (5,000 permutations, adjusted for age, IQ, and same-region GM volumes) (**Table 4**).

**Table 4:** Group difference of fALFF from voxel-wise permutation analysis.

ROI	ANCOVA^a^			Talaraich coordinates		
	p^b^	Bonferroni-corrected p	F^b^	x	y	z
Left DLPFC	0.707	>0.99	67.076	29	53	34
Left insula	0.738	>0.99	16.02	37	26	25
pgACC	0.543	>0.99	36.084	26	47	22
Left SSC	0.353	>0.99	87.685	40	23	37

a Corrected for the effect of age, IQ and same-region GM volume.

b Minimum p and maximum F across all voxels in the corresponding ROI.

### Correlations Between GABA Concentration and Clinical Measurements

Negative correlations of left insula GABA with dyspeptic symptom severity for FD-PDS-MSS (r = −0.49, corrected p = 0.06) and across all patients (r = −0.35, corrected p = 0.06) became insignificant after correction for multiple comparison (**Table 5**). The negative correlation between bilateral pgACC GABA and anxiety symptoms also rendered insignificant after Bonferroni’s correction (r = −0.29, p = 0.19) (**Table 5**).

**Table 5:** Correlations between GABA/water and clinical measures.

MRS ROI	Measure	All subjects (n = 72)			Combined patient group (n = 48)			FD-PDS-MDD (n = 24)			FD-PDS (n = 24)		
		r	p	Bonferroni-corrected p	r	p	Bonferroni-corrected p	r	p	Bonferroni-corrected p	r	p	Bonferroni-corrected p
Left Insula	FGISQ	−0.05	0.66	>0.99	−0.35	0.01	0.06	−0.49	0.01	0.06	−0.23	0.28	>0.99
Bilateral Pregenual Anterior Cingulate	HAMA	−0.11	0.35	>0.99	−0.29	0.05	0.19	−0.34	0.10	0.40	−0.09	0.66	>0.99

MRS ROIs tested: left dorsolateral prefrontal cortex, left insula, bilateral pregenual anterior cingulate, left somatosensory cortex.

Clinical measures tested: FGISQ, Functional Gastrointestinal Standard Questionnaire; BPQ, Body Perception Questionnaire; MADRS, Montgomery–Åsberg Depression Rating Scale; HAMA, Hamilton Anxiety Rating Scale; PHQ15, Patient Health Questionnaire-15; SF36, 36-item Short Form Survey.

No correlations between GABA and health-related quality of life, somatization and introspective awareness were found.

### Correlations Between GABA Concentration and fALFF

In the combined patient group (FD-PDS and FD-PDS-MDD), left insula GABA correlated with same-region fALFF (r = 0.38, corrected p = 0.03) (**Table 6**).

**Table 6:** Correlations between GABA/water and same-region fALFF.

Group	n	MRS ROI	Pearson’s Correlation		
			p	Bonferroni-corrected p	r
All Subjects	72	Left Dorsolateral Prefrontal Cortex	0.92	>0.99	0.01
	72	Left Insula	0.02	0.09	0.27
	72	Bilateral Pregenual Anterior Cingulate	0.47	>0.99	0.09
	72	Left Somatosensory Cortex	0.27	>0.99	−0.13
FD-PDS-MDD	24	Left Dorsolateral Prefrontal Cortex	0.89	>0.99	0.03
	24	Left Insula	0.06	0.24	0.39
	24	Bilateral Pregenual Anterior Cingulate	0.37	>0.99	0.19
	24	Left Somatosensory Cortex	0.33	>0.99	−0.21
FD-PDS	24	Left Dorsolateral Prefrontal Cortex	0.99	>0.99	0.003
	24	Left Insula	0.08	0.32	0.37
	24	Bilateral Pregenual Anterior Cingulate	0.95	>0.99	−0.01
	24	Left Somatosensory Cortex	0.03	0.10	−0.45
Patient	48	Left Dorsolateral Prefrontal Cortex	0.97	>0.99	0.006
	48	Left Insula	0.009	0.03	0.38*
	48	Bilateral Pregenual Anterior Cingulate	0.42	>0.99	−0.12
	48	Left Somatosensory Cortex	0.07	0.26	−0.27

*Bonferroni-corrected p < 0.05.

There were no significant GABA-fALFF correlations in any regions within each patient group or across all subjects.

## Discussion

This is the first study reporting on GABA concentrations in the major somatic-cognitive-affective network in patients with functional gastrointestinal disorders. We failed to find any evidence for altered GABA concentrations in representative regions in the DMN, SN, EN, and SMN in these age/education-matched group of female subjects with FD-PDS with or without comorbid FD-PDS, and healthy subjects. As such, the neurochemical link between FD-PDS and MDD remains elusive.

In measuring GABA concentrations, we used MEGAPRESS which is the standard tool in resolving GABA and with which significant differences had been detected in the DLFPC, pgACC, insula, and somatosensory cortices in (52) patients with various psychiatric conditions using comparable parameters (53–56). We only included subjects with good spectral quality into the analysis; therefore, poor spectral quality, which would have ensued from poor voxel placement, was unlikely to explain the unequivocal findings.

This could be a case of type-II error, although sample size calculation based on previous depression study (GABA = 0.89 (sd = 0.11) in MDD, 1.0 (sd = 0.11) in controls, Cohen’s d = 1.0) showed that 17 subjects were already sufficient to achieve a power of 0.8 with type I error = 0.05 (19). However, the dyspeptic subjects had largely mild dyspeptic severity and for the comorbid MDD group, all met DSM criteria for MDD but also with mild-to-moderate severity of depressive symptoms. While, in lieu of precedence, no comparison can be made with GABA concentrations in other studies on FD, our comorbid FD-MDD did not show changes in GABA in SN (26), DMN (19, 20), and EN (22, 25) reported in previous studies on depressed patients.

Although we used rigorous recruitment assessment for DSM-IV-TR MDD, and Rome III FD-PDS, with negative upper endoscopy results in all recruited dyspeptic subjects to minimize recruitment error, by excluding subjects on centrally-acting drugs we might have recruited subjects with milder symptoms, which may not represent the full spectrum of clinical severity we usually see in our patients. GABA deficit may be obvious only in more clinically severe patients. In order to elucidate the role of GABA in the FD/MDD comorbidity, it is likely that a much larger sample size would be required.

We found, only in depressed dyspeptic subjects, subsignificant negative correlation of GABA concentration in left insula with dyspeptic symptoms. This would require examination in a larger sample to ascertain if SN GABA deficit actually contributed to somatic symptoms, as previous studies on depression suggested (22). However, the weakly positive correlation between GABA concentration with fALFF contradicted the possibility of GABA deficit driving these symptoms by reduction of its inhibitory drive (23, 57). Further examination in larger, well-defined clinical samples would help test against the possibility of spurious associations linked to multiple testing and small sample sizes.

Even more difficult to explain were the unequivocal findings in ALFF from resting state fMRI in all of the regions of interest. This contradicted with two previous reports (27, 28) showing significant increase in SN and DMN resting activity in FD. It is unclear if the difference was attributed to the sample sizes in the dyspeptic comparison arms (40 in Zeng et al., 49 in Liu et al., both from the same group of investigators). Nevertheless, earlier PET-CT studies were not in agreement on resting state findings. One research group in Sichuan reported increased resting activity in pgACC, insula, SSC, and thalamus in FD-PDS patients, positively correlating with dyspeptic symptom severity (27), while the Leuven group reported reduced somatosensory, insula, and cingulate resting glucose metabolism (29). Notwithstanding the fine rigor in these experiments, reports on resting state fMRI changes in FD have remained sparse. Worryingly, after the publication of a number of highly influential studies earlier in the past decade, few if any further neuroimaging studies on FD have been published in the recent few years.

If we accept the validity of our results, their conflict with existing reports may be reconcilable with the complex gut/brain origins of FD and the vast heterogeneity of its pathophysiology across different patients. This may not be entirely surprising if randomized controlled trials of central serotonergic agents have so far reported conflicting results (7, 58, 59). Replication of the previously reported resting state activity findings on larger, cross-national samples including patients of different sociodemographic characteristics and clinical profiles and subsequently, meta-analytic examination will be warranted.

Apart from sample size and potential recruitment bias as mentioned above, our investigation also suffered from a few limitations. Firstly, we did not include a group of MDD-only subjects with no FD and low on somatic symptom scores. Given that somatic symptoms are common in MDD and are even considered a marker of depressive severity (60–63), it would be hard to presume that MDD-only patients without somatic symptoms such as dyspepsia would have more abnormal brain GABA and resting state activity findings. Secondly, we only examined resting-state activity but did not examine brain activations to gastric stimuli. Although significant correlation of anterior cingulate resting activity with regional GABA concentration has been demonstrated (57), GABA was linked to altered task-dependent insula activation in an interoceptive awareness paradigm (26). It should be of interest to examine the link of GABA with task-dependent activation in FD and MDD (29). Thirdly, owing to time constraints, we only included voxels on the left side of the brain (apart from pgACC which was bilateral) considering the vast literature reporting left EN underactivity in major depression (64), but considering the bilateral involvement of prefrontal resting activity reported in FD (27), including right-sided DLPFC, insula and somatosensory voxels may reveal different GABA-resting activity interactions in the FD-MDD comorbidity. Lastly, the present study was planned for comparison of regional GABA concentration and their respective correlation with resting-state activity measure (fALFF). A larger sample size, would be preferred for examination of the correlation of regional GABA concentration with functional connectivity measures in the various different networks owing to the increased number of comparisons involved (57).

In summary, we were unable to identify significant GABA alterations or any contingent changes in resting brain activity in FD-PDS nor could we identify any impact of the FD-depressive comorbidity on neural activity and brain GABA concentrations in the major affective-cognitive-somatosensory networks. FD is a complex and heterogeneous disease of the brain-gut axis (2) with a link to depression—from etiology to treatment—that is fascinating and intriguing. It is possible that the main cortical neurochemical disturbance in FD is glutamatergic (32). A study with larger sample size to delineate the stimulus-dependent activity/glutamate-GABA interactions in the anterior cingulate and bilateral brain networks on patients of a more representative spectrum of clinical severity is clearly indicated. Until then, the foundations for FD to hold claim as a brain-gut disorder will remain uncertain.

## Data Availability Statement

The datasets involved in the study are available from the corresponding author upon reasonable request.

## Ethics Statement

The studies involving human participants were reviewed and approved by New Territories East Cluster- Chinese University of Hong Kong Clinical Research Ethics Committee. The patients/participants provided their written informed consent to participate in this study.

## Author Contributions

AM: study concept and design, study supervision, analysis and interpretation of data, and drafting of manuscript. JH: data analysis, recruitment and behavioral data collection. OL: manuscript drafting. IC: image data processing and analysis. RL: recruitment, medical assessment, and endoscopic evaluation. SW: recruitment, medical assessment, and endoscopic evaluation. DY: image acquisition, technical support. WC: image acquisition, data processing, protocol design and advice on analysis. RE: design of MRS acquisition and voxel placement, image acquisition, data processing, and initial analysis. SC: study conception and design. LL: study conception and design. JW: study concept and design, critical revision of the manuscript for important intellectual content, and study supervision.

## Conflict of Interest

The authors declare that the research was conducted in the absence of any commercial or financial relationships that could be construed as a potential conflict of interest.
